# Exhaustive data mining comparison of the effects of low doses of ionizing radiation, formaldehyde and dioxins

**DOI:** 10.1186/1471-2164-15-S12-S5

**Published:** 2014-12-19

**Authors:** Alexey Moskalev, Mikhail Shaposhnikov, Ekaterina Plyusnina, Sergey Plyusnin, Olga Shostal, Alexander Aliper, Alex Zhavoronkov

**Affiliations:** 1 Institute of Biology, Komi Science Center of Russian Academy of Sciences; Syktyvkar, Russian Federation; 2 Syktyvkar State University, Syktyvkar, Russian Federation; 3 Moscow Institute of Physics and Technology, Moscow, Russian Federation; 4 Federal Clinical Research Center of Pediatric Hematology, Oncology, and Immunology, Moscow, Russian Federation; 5 The Biogerontology Research Foundation, London, UK

## Abstract

**Background:**

Ionizing radiation in low doses is the ubiquitous environmental factor with harmful stochastic effects. Formaldehyde is one of the most reactive household and industrial pollutants. Dioxins are persistent organic pollutants and most potent synthetic poisons effective even at trace concentrations. Environmental pollutants are capable of altering the expression of a variety of genes. To identify the similarities and differences in the effects of low-dose ionizing radiation, formaldehyde and dioxin on gene expression, we performed the bioinformatic analysis of all available published data.

**Results:**

We found that that in addition to the common p53-, ATM- and MAPK-signaling stress response pathways, genes of cell cycle regulation and proinflammatory cytokines, the studied pollutants induce a variety of other molecular processes.

**Conclusions:**

The observed patterns provide new insights into the mechanisms of the adverse effects associated with these pollutants. They can also be useful in the development of new bio-sensing methods for detection of pollutants in the environment and combating the deleterious effects.

## Background

Regardless of their chemical and physical nature, all stressors influence organisms by changing the cell functioning. This is achieved through alterations in the genome function that manifest themselves by changes in the expression and activity of certain genes [1-9]. Several avenues are available for a stressor to influence the gene expression. It can be achieved directly through the damaging of gene's DNA, indirectly through the mechanisms of damage detection followed by the induction of stress response, or by direct action of stressor on the components of intracellular signaling machinery (cell receptors, transcription factors, kinases) [10,11].

The term "genotoxicants" refers to the factors that are capable of inflicting the damage to DNA molecules. DNA is the most vulnerable among all cellular structures. By coding all proteins the cell needs, DNA orchestrates the cellular activity. However, a single cell possesses only two copies of each DNA molecule. While other damaged macromolecules such as proteins, lipids and carbohydrates may be replaced by intact copies, the DNA damage can lead to disastrous consequences. By causing inheritable changes in the generations of cells and organisms, genotoxic agents affect the incidence of human diseases and biodiversity of biota [12,13]. They cause heritable adverse effects among the offspring, increase the rate of cancer development and accelerate aging [14,15].

In response to the damage of DNA or other cellular structures, the stress response based on the changes in the level of expression of certain genes gets generated [7,8]. For certain genes this may be an increase in the activity while for others the activity diminishes. Some of these changes have a protective, adaptive character, while others are the result of the genome dysfunction (genotoxic effect). It can be assumed that adaptive changes have deterministic and reproducible nature since they were formed as a result of long evolution of the stress response. The effects of genome malfunctioning are stochastic in nature: they depend on the locus of damaged DNA, its position in euchromatin or heterochromatin regions, the importance of the damaged gene for the functioning of certain cell type during certain period of ontogenesis, and the number and extent of the lesions.

Recently we have studied genome-wide transcriptional response to ionizing radiation, formaldehyde, toluene, and 2,3,7,8-tetrachlorodibenzo-*p*-dioxin exposure on *Drosophila melanogaster *whole-animal model [7]. The RNA-seq analysis on 25,415 transcripts revealed both significant similarities and differences in differential gene expression and the activity of biological processes under the influence of each treatment. Some of the observed transcriptional changes in stress can be regarded as protective and adaptive in nature (cell cycle arrest, induction of antioxidant and DNA repair systems, molecular chaperones), while the rest are related to the dysfunction of cellular systems (violation of redox and biosynthetic processes) [7]. The transcriptome changes in response to all the studied types of stresses involve differential regulation of a large common cluster of the genes, most of them earlier identified as related to genome maintenance or aging.

In another recent work, Brown et al. studied the transcriptional effects of environmental perturbations (cold, heat, caffeine, paraquat, rotenone, copper, zinc, and cadmium) in Drosophila model [8]. They found a uniform response to environmental stressors. The changes in the activity of most genes is reproduced after most of studied treatments [8]. The unregulated genes included those annotated with the GO term ‘‘Response to Stimulus, GO:0050896’’, and those that encode lysozymes, cytochrome P450s, and mitochrondrial components mt:ATPase6, mt:CoI, mt:CoIII. The downregulated genes encoded egg-shell, yolk and seminal fluid proteins [8].

In addition, environmental pollutants may influence the intracellular signaling machinery that mediates the regulation of gene expression. For example, ionizing radiation is capable of causing the formation of reactive oxygen species which damage various proteins including regulatory ones [16]. Formaldehyde promotes formation of protein-protein and DNA-protein crosslinks [17]. 2,3,7,8-Tetrachlorodibenzo-*p*-dioxin is not a direct genotoxicant but it binds to an intracellular protein, aryl hydrocarbon receptor (AhR) [18]. The latter is a transcriptional enhancer that influences expression of some key cellular genes.

Development of new effective test systems for the detection of environmental mutagens at low concentrations is an important practical task. Biosensor is a biological detector (a particular molecule, cell or tissue) that can respond, in a predictable manner, to the investigated factor (chemical compound or physical action). Currently, the measurements of the damage level are widely used for the purposes of revealing the effects of environmental pollutants. Commonly used damage indicators include the number of micronuclei in the bone marrow of animals, anaphase bridges and fragments, and the proportion of damaged DNA determined by the DNA comet assay The methods of genetic analysis, however, are labor-intensive. Identification of adaptive changes in the gene expression may be a more reliable and less time consuming way of bio-sensing of damaging effects compared to the measurement of stochastic damages, particularly at low concentrations (or doses) of damaging factor.

From the bio-sensing point of view, the small doses of ionizing radiation, formaldehyde and dioxins are some of the most relevant environmental factors. They are important due to their prevalence and the risk of long-term effects. The purpose of this study was to identify the similarities and differences in effects of low doses of ionizing radiation, formaldehyde and dioxin on the expression of genes in different mammalian species (mouse, rat, human). The data on the gene expression are among the most important objects of study of the modern bioinformatics. The bioinformatics analysis conducted in this work can become the basis for new methods of the pollutants bio-sensing in the environment, in particular for the establishment of biosensor expression chips (RNA microarrays) or PCR sets (PCR-arrays).

## Results

Additional file 1 Table S1 combines the literature data on the genes that get activated in response to low doses of radiation, formaldehyde and dioxins. As the table shows, only a small proportion of these genes overlap and get activated by several different exposures. Genes TRP53, CDKN1A and AREG are activated under the influence of both ionizing radiation and formaldehyde. Induction of genes CDKN1A, BAX, AREG, EGR1 and TNF is observed under the influence of both radiation and dioxin. Dioxin and formaldehyde can both cause expression of genes CDKN1A and AREG. Only two genes from the list, CDKN1A and AREG, respond to all three types of pollutants. At the same time, the analysis of gene ontology annotated for presented genes shows a much more significant overlapping in the functions of these genes. Two hundred gene ontologies are common for all genes and influences. A considerable number of other ontologies overlap within the pairs of analyzed influences. In particular, 210 common biological processes were observed for the effects of dioxin and radiation, 101 - for radiation and formaldehyde, 47 - for formaldehyde and dioxin. Comparison of the number of genes involved in a particular process during different influences (Additional file 2 Table S2 and Additional file 3 Table S3) shows that all three analyzed pollutants substantially activate p53, ATM and MAPK stress response signaling pathways, cell cycle regulating genes, and the production of pro-inflammatory cytokines.

The differences in the effects of investigated pollutants are as interesting as their common features. Radiation increases the expression of cell differentiation genes and genes involved in apoptosis and response to DNA damage. It causes stress induction of heat shock proteins and cellular senescence (Additional file 3 Table S3). Dioxin induces the metabolism of xenobiotics and drugs by cytochrome P450, metabolism of retinol and tryptophan, as well as chemical carcinogenesis. It stimulates oxidative stress through the transcription factor Nrf2. It also influences the hematopoiesis process (Additional file 2 Table S2). Formaldehyde influenced the genes of the circadian cycle and stress kinase p38, caused the endoplasmic reticulum stress and G2/M cell cycle checkpoint (Additional file 2 Table S2 and Additional file 3 Table S3). Interestingly, the adverse factors studied in this work were activating the genes involved in the development of various neoplastic processes and certain diseases such as rheumatoid arthritis, hepatitis C and amyotrophic lateral sclerosis (Additional file 2 Table S2). Thus, the pollutants are able to promote pathologies, contribute to their development or cause them in the first place (as in the case of tumors).

**Figure 1:**
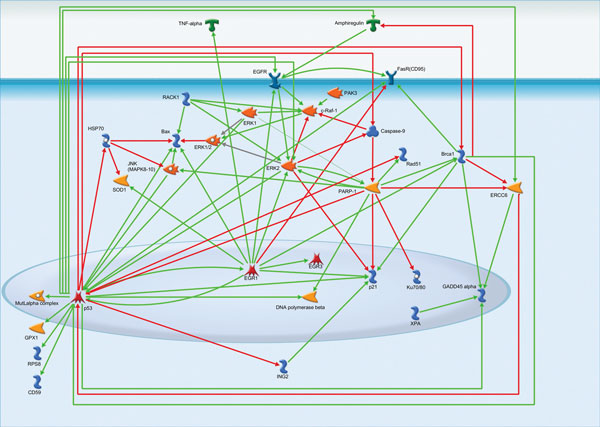
**Interactions between the activated gene products in ionizing radiation group**. For figures 1 to 3: The activation and inhibition interactions between proteins are shown using green and red arrows respectively. Group relationships between proteins are depicted with grey arrows.

The analysis of interactions between the products of activated genes revealed that in ionizing radiation group the EGF Receptor may be activated by Amphiregulin protein. In turn, EGF receptor conveys the signal towards Fas receptor, c-Raf-1 and ERK2. ERK2 could possibly transmit the signal to p53 and DNA polymerase beta via activation of PARP-1. P53 stands as the most interconnected gene in this group. It activates another transcriptional factor EGR1 that up-regulates SOD1, Bax, TNF-alpha, EGFR, ERK2, EGR3 and p21. EGR1 is one of the most connected elements in the network and is very involved in stress response. The p53-EGR1 duet could possibly serve as a trigger for the response to ionizing radiation (Figure 1).

**Figure 2:**
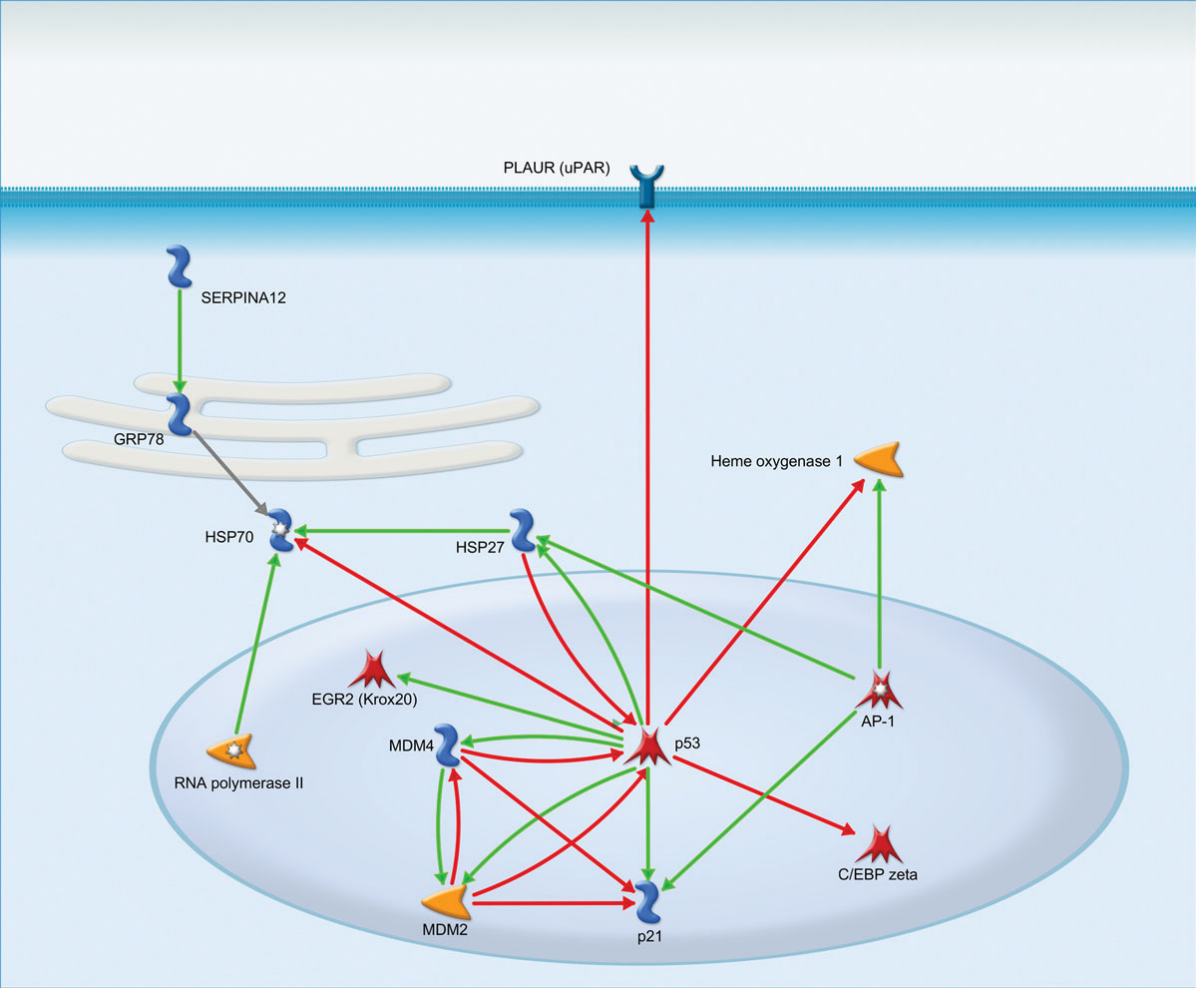
**Interactions between the gene products in formaldehyde group**.

In formaldehyde-activated gene group, p53 also is the most interconnected node of the network (Figure 2). It activates HSP27, EGR2, MDM4, MDM2, p21 and inhibits urokinase receptor, heme oxygenase 1, C/EBP zeta and HSP70. Activator protein 1 (AP-1) also activates HSP27 and p21, but unlike p53 it activates Heme oxygenase 1. Upregulated RNA polymerase II engages in heat shock response by activating HSP70 which may be also activated by serpin peptidase inhibitor (SERPINA12).

**Figure 3:**
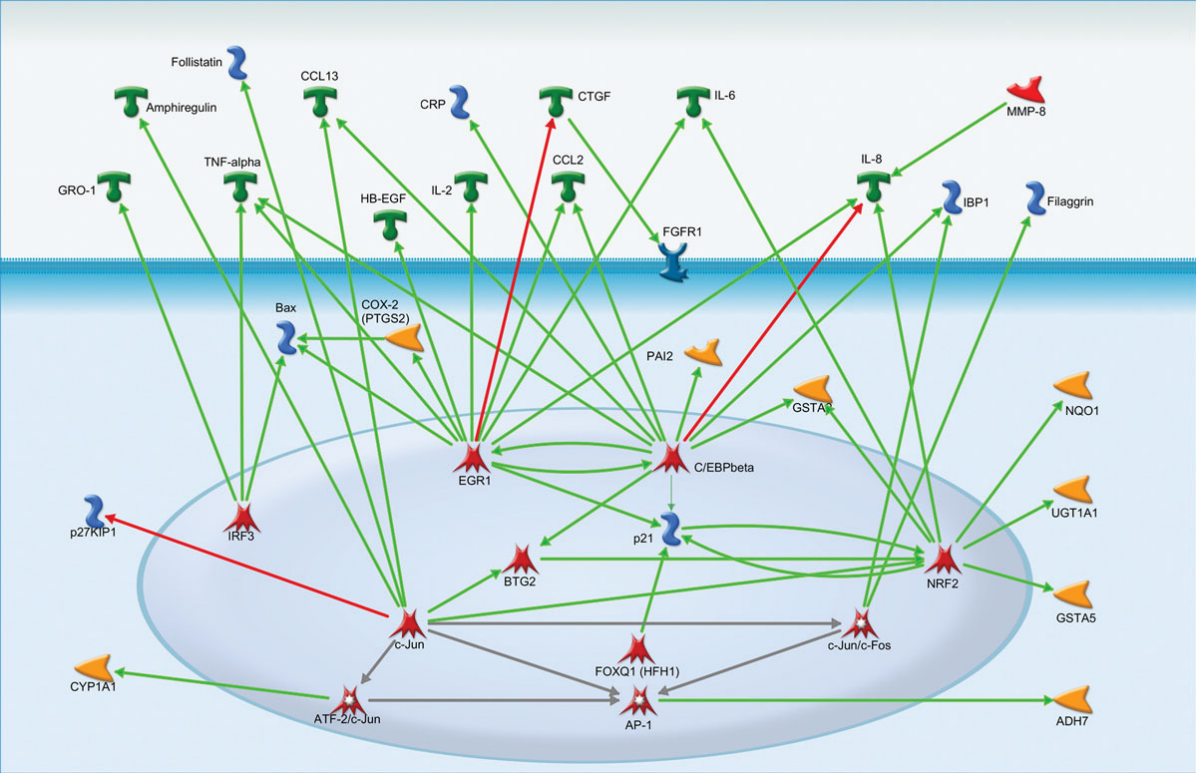
**Interactions between the gene products in dioxin group**.

In case of gene group activated by Dioxin exposure we can see significant upregulation of various ligands. This could be explained by the action of several transcriptional factors: IRF3, c-Jun, EGR1, C/EBPbeta (Figure 3).

## Discussion

The obtained bioinformatics analysis data point to the induction of both common and different molecular processes by low-dose ionizing radiation, formaldehyde and dioxin. The similarity of ionizing radiation and formaldehyde effects on gene expression was also observed in our recent investigation performed on *Drosophila *[7]. The most similar set of changes revealed in *Drosophila *for 'dioxin and radiation' is also confirmed by bioinformatics analysis [7]. The homogeneous response to different kind of environmental perturbations, such as cold, heat, caffeine, paraquat, rotenone, copper, zinc, and cadmium was also found by Brown et al. in *Drosophila *model [8].

The activation of stress response genes after exposure to ionizing radiation and pollutants can cause or aggravate the development of various chronic diseases on the organismal level. Moreover recently we have demonstrated that the majority of stress response genes are highly interconnected and may cause longevity or aging depending on the exposure dose [19].

The differences in the spectrum of expressed genes induced by different factors can serve as a basis for the development of new methods of revealing of the effects of environmental pollutants. These methods could be based on bio-sensing of impact through quantifying the mRNAs of suitable genes by RT-PCR, expression chips or RNA-Seq. The transgenic organisms with green fluorescent protein (GFP) gene expression driven by the promoter of unique genes may be used for detection of low doses of ionizing radiation and pollutants.

## Conclusion

Thus the observed patterns of changes in gene expression levels provide new insights into the mechanisms of the deleterious effects of the exposure to ionizing radiation and chemical pollutants. These data can also be used for bio-sensing of pollutants in the environment and combating the adverse effects.

## Methods

### Generation of the lists of genes that increase expression in response to the ionizing radiation, formaldehyde and dioxin exposure

Gene lists were obtained by analysis of the literature that provides experimental data on the effects of stressors on the expression of mammalian genes (human cells, mouse and rat). Using the Entrez gene database http://www.ncbi.nlm.nih.gov/gene, the resulting lists have been brought in line with the official names of the genes in the mouse genome.

According to the used publications the ionizing radiation absorbed dose rate was ranged from 0.1 to 10 cGy, that corresponds to the low dose range of low-LET radiation [20]. The concentration of TCDD was 0.2-10 nM. Concentration of formaldehyde was 40-200 µM in cell culture media, or 0.7-15 ppm in air (for animal experiments).

### Bioinformatics analysis of genes function

All procedures for analysis and comparison of gene lists were performed in the statistical programming environment R (version 2.15.3). Molecular process annotation-based description of each exposure was executed on the basis of analysis of the number of listed genes belonging to a particular category. The level of significance of P-value and FDR amendment (False Discovery Rate control) were taken into account [21].

To analyze the functions of considered genes, a "gene ontology" (GO) was used. GO is a project in the field of bioinformatics devoted to unify the attributes of genes and gene products of all species [22]. The objective of the project is to make annotations to the genes and products, and maintain and update a clearly defined list of attributes of genes and their products according to the categories of "biological processes" "biological functions" and "structural components." Getting the gene ontology for the lists of considered genes was performed with the use of R package BioMart [23,24]. Analysis of the gene ontologies overlapping for different influencing factors was carried out in the R package VennDiagram [25]. Statistical significance and visualization of gene ontologies for different influences were presented in the form of a "word cloud" in the R package GOsummaries [26].

In addition to the analysis of gene ontologies, the comparisons by KEGG and caBIO were made to compare the functional characteristics of genes that get activated at different exposures. KEGG is a molecular pathways annotation method which involved a particular gene proposed by the biological information resource KEGG (Kyoto Encyclopedia of Genes and Genome http://www.genome.jp/kegg). caBIO (Cancer Bioinformatics Infrastructure Objects) is a similar project run by the NCI https://wiki.nci.nih.gov/display/caBIO. Analysis and comparison of the investigated influencing factors by means of molecular mechanisms annotations offered by KEGG and caBIO were made in the R package GeneAnswers [27].

To analyze the interactions of protein products of activated genes in each group of environmental pollutants we utilized Thomson Reuters MetaCore™ service http://thomsonreuters.com/metacore/. In every group we looked at all scientifically documented interactions between the gene products.

## Competing interests

The authors declare that they have no competing interests.

## Authors' contributions

AM, MS, EP, SP, OS, AA, AZ wrote the manuscript text. AM and AA carried out the bioinformatic analysis. AA prepared the figures. AM and AZ supervised the bioinformatic research and text of the manuscript. All authors read and approved the final manuscript.

## Supplementary Material

Additional file 1 **Table S1**. Genes activated in response to the radiation exposure, formaldehyde and dioxins. Click here for file

Additional file 2 **Table S2**. Comparison of the number of genes involved in various molecular processes (according to KEGG) that increase activity under the influence of different pollutants. Click here for file

Additional file 3 **Table S3**. Comparison of the number of genes involved in various molecular processes (according to caBIO) that increase activity under the influence of different pollutants. Click here for file

## Declarations

Publication of this article has been funded by the Biogerontology Research Foundation.

This article has been published as part of *BMC Genomics *Volume 15 Supplement 12, 2014: Selected articles from the IX International Conference on the Bioinformatics of Genome Regulation and Structure\Systems Biology (BGRS\SB-2014): Genomics. The full contents of the supplement are available online at http://www.biomedcentral.com/bmcgenomics/supplements/15/S12.
