# Supplementary material for: Exhaustive data mining comparison of the effects of low doses of ionizing radiation, formaldehyde and dioxins
Source: BMC Genomics. 2014 Dec 19;15(Suppl 12):S5. doi: 10.1186/1471-2164-15-S12-S5 (PMC4303946; doi:10.1186/1471-2164-15-S12-S5)
Supplement: Additional file 1 — Table S1. Genes activated in response to the radiation exposure, formaldehyde and dioxins. [file 1471-2164-15-S12-S5-S1.doc]

**Table S1 Genes activated in response to the radiation exposure, formaldehyde and dioxins***

| **Pollutants** | **Activated genes** | **References** |
| --- | --- | --- |
| Ionizing radiation | *GPX1, SOD1, GADD45A,* ***TRP53****, MAPK8,* ***CDKN1A****, GNB2L1, BRCA1, PARP1, POLB, ERCC6, XPA, MLH1, RAD51, XRCC5,* ***BAX****, MAPK1, CASP9, FAS, ING2, LTBP1,* ***AREG****, MOS, RAF1, AGGF1, EGFR,* ***EGR1****, CD59A, EGR3,* ***TNF****, RAB6A, Zeb2, SMGC, GJC1, EIF3E, RPL24, RPS8, OAZ1, CCT6A, KLK1B21, KLK1B4, HSPA1B, HSPA8, ELOVL5, G6PC, ATP10D, ATP1B2, ACTR1A* |  |
| Formaldehyde | *HSPA5, DDIT3, TXN1, IL4, IFNG, PRDX1,* ***TRP53****,* ***CDKN1A****, MDM2, MDM4, HMOX1, TNFRSF12A, TXNRD1, FOSL1, POLR2D, PLAUR, SRXN1, HSPB1, MAP3K8, PTHLH,* ***AREG****, EGR2, PVR, ATRX, SLC25A15, ARHGAP5, PER2, SERPINA12, PUM1, ARNTL, GALNT7, PLA2G4A, MAP3K1, SEC22A* |  |
| Dioxin | *ADH7, CYP1A1, CYP1A2, CYP1B1, UGT1A1, UGT1A6A, NQO1, ALDH3A1, POR, GSTA2, NFE2L2, JUN, JUND,* ***CDKN1A****, FLG, CDKN1B, HESX1, IL2,* ***BAX****, IGFBP1, CLDN25, MICAL2, GANC, ARL6IP5, SPINT1, SERPINB2, TIPARP, 4931440P22RIK, CCL2, CXCL1, CCL1, IL6, PTGS2,* ***TNF****, IL8, PTGS2,* ***TNF****, IL8, CRP, CEBPB, IL17RB, MYOF, SLC7A5, SCIN, MMP8, IL9, FLNB, GM5662, NMT2,* ***EGR1****, AHRR,* ***AREG****, HBEGF, CTGF, FST, BTG2, FOXQ1, TNFSF13B, CXCL13, IRF3* |  |

**Notes:** * Mammalian genes identified during the exposure to these factors in different species (mouse, rat, human). Gene names are aligned according to the names in the mouse genome. Genes that get activated by more than one pollutant are highlighted in bold.

**References**

1. Saini D, Shelke S, Mani Vannan A, Toprani S, Jain V, Das B, Seshadri M: **Transcription profile of DNA damage response genes at G(0) lymphocytes exposed to gamma radiation.** *Mol Cell Biochem* 2012, **364:**271-281.

2. Wyrobek AJ, Manohar CF, Krishnan VV, Nelson DO, Furtado MR, Bhattacharya MS, Marchetti F, Coleman MA: **Low dose radiation response curves, networks and pathways in human lymphoblastoid cells exposed from 1 to 10cGy of acute gamma radiation.** *Mutat Res* 2011, **722:**119-130.

3. Long XH, Zhao ZQ, He XP, Wang HP, Xu QZ, An J, Bai B, Sui JL, Zhou PK: **Dose-dependent expression changes of early response genes to ionizing radiation in human lymphoblastoid cells.** *Int J Mol Med* 2007, **19:**607-615.

4. Rudqvist N, Parris TZ, Schuler E, Helou K, Forssell-Aronsson E: **Transcriptional response of BALB/c mouse thyroids following in vivo astatine-211 exposure reveals distinct gene expression profiles.** *EJNMMI Res* 2012, **2:**32.

5. Lanza V, Pretazzoli V, Olivieri G, Pascarella G, Panconesi A, Negri R: **Transcriptional response of human umbilical vein endothelial cells to low doses of ionizing radiation.** *J Radiat Res* 2005, **46:**265-276.

6. Howell EK, Gaschak SP, Griffith KD, Rodgers BE: **Radioadaptive response following in utero low-dose irradiation.** *Radiat Res* 2013, **179:**29-37.

7. Furlong H, Mothersill C, Lyng FM, Howe O: **Apoptosis is signalled early by low doses of ionising radiation in a radiation-induced bystander effect.** *Mutat Res* 2013, **741-742:**35-43.

8. Albrecht H, Durbin-Johnson B, Yunis R, Kalanetra KM, Wu S, Chen R, Stevenson TR, Rocke DM: **Transcriptional response of ex vivo human skin to ionizing radiation: comparison between low- and high-dose effects.** *Radiat Res* 2012, **177:**69-83.

9. von Neubeck C, Shankaran H, Karin NJ, Kauer PM, Chrisler WB, Wang X, Robinson RJ, Waters KM, Tilton SC, Sowa MB: **Cell type-dependent gene transcription profile in a three-dimensional human skin tissue model exposed to low doses of ionizing radiation: implications for medical exposures.** *Environ Mol Mutagen* 2012, **53:**247-259.

10. Luo FC, Zhou J, Lv T, Qi L, Wang SD, Nakamura H, Yodoi J, Bai J: **Induction of endoplasmic reticulum stress and the modulation of thioredoxin-1 in formaldehyde-induced neurotoxicity.** *Neurotoxicology* 2012, **33:**290-298.

11. Xu B, Aoyama K, Takeuchi M, Matsushita T, Takeuchi T: **Expression of cytokine mRNAs in mice cutaneously exposed to formaldehyde.** *Immunol Lett* 2002, **84:**49-55.

12. Lim SK, Kim JC, Moon CJ, Kim GY, Han HJ, Park SH: **Formaldehyde induces apoptosis through decreased Prx 2 via p38 MAPK in lung epithelial cells.** *Toxicology* 2010, **271:**100-106.

13. Wong VC, Cash HL, Morse JL, Lu S, Zhitkovich A: **S-phase sensing of DNA-protein crosslinks triggers TopBP1-independent ATR activation and p53-mediated cell death by formaldehyde.** *Cell Cycle* 2012, **11:**2526-2537.

14. Andersen ME, Clewell HJ, 3rd, Bermudez E, Willson GA, Thomas RS: **Genomic signatures and dose-dependent transitions in nasal epithelial responses to inhaled formaldehyde in the rat.** *Toxicol Sci* 2008, **105:**368-383.

15. Vogel CF, Li W, Wu D, Miller JK, Sweeney C, Lazennec G, Fujisawa Y, Matsumura F: **Interaction of aryl hydrocarbon receptor and NF-κB subunit RelB in breast cancer is associated with interleukin-8 overexpression.** *Arch Biochem Biophys* 2011, **512:**78-86.

16. Vogel CF, Sciullo E, Matsumura F: **Involvement of RelB in aryl hydrocarbon receptor-mediated induction of chemokines.** *Biochem Biophys Res Comm* 2007, **363:**722-726.

17. Henry EC, Welle SL, Gasiewicz TA: **TCDD and a putative endogenous AhR ligand, ITE, elicit the same immediate changes in gene expression in mouse lung fibroblasts.** *Toxicol Sci* 2010, **114:**90-100.

18. Hao N, Lee KL, Furness SG, Bosdotter C, Poellinger L, Whitelaw ML: **Xenobiotics and loss of cell adhesion drive distinct transcriptional outcomes by aryl hydrocarbon receptor signaling.** *Mol Pharmacol* 2012, **82:**1082-1093.

19. Faust D, Vondráček J, Krčmář P, Smerdová L, Prochazkova J, Hruba E, Hulinkova P, Kaina B, Dietrich C, Machala M: **AhR-mediated changes in global gene expression in rat liver progenitor cells.** *Arch Toxicol* 2013, **87:**681-698.

20. Le Vee M, Jouan E, Fardel O: **Involvement of aryl hydrocarbon receptor in basal and 2,3,7,8-tetrachlorodibenzo-*p*-dioxin-induced expression of target genes in primary human hepatocytes.** *Toxicol In Vitro* 2010, **24:**1775-1781.

21. Yueh MF, Huang YH, Hiller A, Chen S, Nguyen N, Tukey RH: **Involvement of the xenobiotic response element (XRE) in Ah receptor-mediated induction of human UDP-glucuronosyltransferase 1A1.** *J Biol Chem* 2003, **278:**15001-15006.

22. Münzel PA, Lehmköster T, Brück M, Ritter JK, Bock KW: **Aryl hydrocarbon receptor-inducible or constitutive expression of human UDP glucuronosyltransferase UGT1A6.** *Arch Biochem Biophys* 1998, **350:**72-78.

23. Münzel PA, Schmohl S, Buckler F, Jaehrling J, Raschko FT, Köhle C, Bock KW: **Contribution of the Ah receptor to the phenolic antioxidant-mediated expression of human and rat UDP-glucuronosyltransferase UGT1A6 in Caco-2 and rat hepatoma 5L cells.** *Biochem Pharmacol* 2003, **66:**841-847.

24. Rushmore TH, Pickett CB: **Transcriptional regulation of the rat glutathione S-transferase Ya subunit gene. Characterization of a xenobiotic-responsive element controlling inducible expression by phenolic antioxidants.** *J Biol Chem* 1990, **265:**14648-14653.

25. Miao W, Hu L, Scrivens PJ, Batist G: **Transcriptional regulation of NF-E2 p45-related factor (NRF2) expression by the aryl hydrocarbon receptor-xenobiotic response element signaling pathway: direct cross-talk between phase I and II drug-metabolizing enzymes.** *J Biol Chem* 2005, **280:**20340-20348.

26. Hoffer A, Chang CY, Puga A: **Dioxin induces transcription of *fos* and *jun* genes by *Ah* receptor-dependent and -independent pathways.** *Toxicol Appl Pharmacol* 1996, **141:**238-247.

27. Barnes-Ellerbe S, Knudsen KE, Puga A: **2,3,7,8-Tetrachlorodibenzo-*p*-dioxin blocks androgen-dependent cell proliferation of LNCaP cells through modulation of pRB phosphorylation.** *Mol Pharmacol* 2004, **66:**502-511.

28. Loertscher JA, Lin TM, Peterson RE, Allen-Hoffmann BL: ***In utero* exposure to 2,3,7,8-tetrachlorodibenzo-*p*-dioxin causes accelerated terminal differentiation in fetal mouse skin.** *Toxicol Sci* 2002, **68:**465-472.

29. Kolluri SK, Weiss C, Koff A, Göttlicher M: **p27^Kip1^ induction and inhibition of proliferation by the intracellular Ah receptor in developing thymus and hepatoma cells.** *Genes Dev* 1999, **13:**1742-1753.

30. Thomsen JS, Kietz S, Strom A, Gustafsson JA: **HES-1, a novel target gene for the aryl hydrocarbon receptor.** *Mol Pharmacol* 2004, **65:**165-171.

31. Jeon MS, Esser C: **The murine IL-2 promoter contains distal regulatory elements responsive to the Ah receptor, a member of the evolutionarily conserved bHLH-PAS transcription factor family.** *J Immunol* 2000, **165:**6975-6983.

32. Matikainen T, Perez GI, Jurisicova A, Pru JK, Schlezinger JJ, Ryu HY, Laine J, Sakai T, Korsmeyer SJ, Casper RF, et al: **Aromatic hydrocarbon receptor-driven *Bax* gene expression is required for premature ovarian failure caused by biohazardous environmental chemicals.** *Nat Genet* 2001, **28:**355-360.

33. Marchand A, Tomkiewicz C, Marchandeau JP, Boitier E, Barouki R, Garlatti M: **2,3,7,8-Tetrachlorodibenzo-*p*-dioxin induces insulin-like growth factor binding protein-1 gene expression and counteracts the negative effect of insulin.** *Mol Pharmacol* 2005, **67:**444-452.

34. Gao L, Dong L, Whitlock JP, Jr.: **A novel response to dioxin. Induction of ecto-ATPase gene expression.** *J Biol Chem* 1998, **273:**15358-15365.

35. Vogel CF, Nishimura N, Sciullo E, Wong P, Li W, Matsumura F: **Modulation of the chemokines KC and MCP-1 by 2,3,7,8-tetrachlorodibenzo-p-dioxin (TCDD) in mice.** *Arch Biochem Biophys* 2007, **461:**169-175.

36. N'Diaye M, Le Ferrec E, Lagadic-Gossmann D, Corre S, Gilot D, Lecureur V, Monteiro P, Rauch C, Galibert MD, Fardel O: **Aryl hydrocarbon receptor- and calcium-dependent induction of the chemokine CCL1 by the environmental contaminant benzo[a]pyrene.** *J Biol Chem* 2006, **281:**19906-19915.

37. Vogel CF, Sciullo E, Wong P, Kuzmicky P, Kado N, Matsumura F: **Induction of proinflammatory cytokines and C-reactive protein in human macrophage cell line U937 exposed to air pollution particulates.** *Environ Health Perspect* 2005, **113:**1536-1541.

38. Casado FL, Singh KP, Gasiewicz TA: **Aryl hydrocarbon receptor activation in hematopoietic stem/progenitor cells alters cell function and pathway-specific gene modulation reflecting changes in cellular trafficking and migration.** *Mol Pharmacol* 2011, **80:**673-682.

39. Kolluri SK, Balduf C, Hofmann M, Gottlicher M: **Novel target genes of the Ah (dioxin) receptor: transcriptional induction of N-myristoyltransferase 2.** *Cancer Res* 2001, **61:**8534-8539.

40. Martinez JM, Baek SJ, Mays DM, Tithof PK, Eling TE, Walker NJ: **EGR1 is a novel target for AhR agonists in human lung epithelial cells.** *Toxicol Sci* 2004, **82:**429-435.
