# Supplementary material for: Exhaustive data mining comparison of the effects of low doses of ionizing radiation, formaldehyde and dioxins
Source: BMC Genomics. 2014 Dec 19;15(Suppl 12):S5. doi: 10.1186/1471-2164-15-S12-S5 (PMC4303946; doi:10.1186/1471-2164-15-S12-S5)
Supplement: Additional file 2 — Table S2. Comparison of the number of genes involved in various molecular processes (according to KEGG) that increase activity under the influence of different pollutants. [file 1471-2164-15-S12-S5-S2.doc]

**Table S2 Comparison of the number of genes involved in various molecular processes (according to KEGG) that increase activity under the influence of different pollutants***

| **KEGG Terms** | **Rad** | **For** | **Dio** |
| --- | --- | --- | --- |
| p53 signaling pathway | **6** | **3** | 2 |
| MAPK signaling pathway | **9** | **6** | 4 |
| ErbB signaling pathway | 5 | 2 | **5** |
| Metabolism of xenobiotics by cytochrome P450 | 0 | 0 | **7** |
| Drug metabolism - cytochrome P450 | 0 | 0 | **5** |
| Retinol metabolism | 0 | 0 | **4** |
| Tryptophan metabolism | 0 | 0 | **3** |
| Cell cycle | 3 | **3** | 2 |
| Circadian rhythm - mammal | 0 | **2** | 0 |
| Cytokine-cytokine receptor interaction | 3 | 3 | **9** |
| Intestinal immune network for IgA production | 0 | 1 | **3** |
| T cell receptor signaling pathway | 3 | **3** | 3 |
| Hepatitis C | **7** | 2 | 3 |
| Amyotrophic lateral sclerosis (ALS) | **6** | 1 | 2 |
| Prion diseases | 4 | 1 | **3** |
| Rheumatoid arthritis | 1 | 1 | **4** |
| Chagas disease (American trypanosomiasis) | 3 | 1 | **4** |
| Endometrial cancer | **6** | 1 | 0 |
| Colorectal cancer | **6** | 1 | 2 |
| Pancreatic cancer | **6** | 1 | 0 |
| Pathways in cancer | **10** | 3 | 6 |
| Bladder cancer | **5** | **3** | 1 |
| Prostate cancer | **6** | **3** | 2 |
| Glioma | 5 | **3** | 1 |
| Melanoma | 5 | **3** | 1 |
| Chronic myeloid leukemia | 4 | **3** | 2 |
| Total number of genes | 48 | 34 | 56 |

**Notes:** * Statistically significant differences are highlighted in bold, Rad - ionizing radiation, For - formaldehyde, Dio - dioxin.
