# Supplementary material for: Exhaustive data mining comparison of the effects of low doses of ionizing radiation, formaldehyde and dioxins
Source: BMC Genomics. 2014 Dec 19;15(Suppl 12):S5. doi: 10.1186/1471-2164-15-S12-S5 (PMC4303946; doi:10.1186/1471-2164-15-S12-S5)
Supplement: Additional file 3 — Table S3. Comparison of the number of genes involved in various molecular processes (according to caBIO) that increase activity under the influence of different pollutants. [file 1471-2164-15-S12-S5-S3.doc]

**Table S3 Comparison of the number of genes involved in various molecular processes (according to caBIO) that increase activity under the influence of different pollutants***

| **CABIO.PATH Terms** | **Rad** | **For** | **Dio** |
| --- | --- | --- | --- |
| ATM Signaling Pathway | **5** | **3** | 2 |
| p53 Signaling Pathway | **4** | **3** | 2 |
| Free Radical Induced Apoptosis | **3** | 0 | 1 |
| Apoptotic Signaling in Response to DNA Damage | **4** | 1 | 1 |
| Keratinocyte Differentiation | **5** | 1 | 2 |
| Cell Cycle: G2/M Checkpoint | **4** | **3** | 1 |
| Phosphorylation of MEK1 by cdk5/p35 down regulates the MAP kinase pathway | **3** | 0 | 1 |
| Stress Induction of HSP Regulation | **3** | 1 | 1 |
| Cadmium induces DNA synthesis and proliferation in macrophages | **3** | 0 | 2 |
| Telomeres, Telomerase, Cellular Aging, and lmmortality | **3** | 1 | 0 |
| The 4-1BB-dependent immune response | 0 | **3** | 2 |
| p38 МАРК Signaling Pathway | 0 | **3** | 0 |
| Oxidative Stress Induced Gene Expression Via Nrf2 | 1 | 1 | **4** |
| Cytokine Network | 1 | 2 | **4** |
| Regulation of hematopoiesis by cytokines | 0 | 1 | **3** |
| Mechanism of Acetaminophen Activity and Toxicity | 0 | 0 | **2** |
| Msp/Ron Receptor Signaling Pathway | 1 | 0 | **2** |
| IL 6 signaling pathway | 1 | 0 | **3** |
| Cytokines and Inflammatory Response | 1 | 2 | **3** |
| The information-processing pathway at the IFN-beta enhancer | 0 | 0 | **2** |
| Total number of genes | 48 | 34 | 56 |

**Notes:** * Statistically significant differences are highlighted in bold, Rad - ionizing radiation, For - formaldehyde, Dio - dioxin.
